# Single-cell transcriptome analysis of uncultured human umbilical cord mesenchymal stem cells

**DOI:** 10.1186/s13287-020-02055-1

**Published:** 2021-01-07

**Authors:** Shaoyang Zhang, Jing Yi Wang, Baojie Li, Feng Yin, Huijuan Liu

**Affiliations:** 1 Bio-X Institutes, Key Laboratory for the Genetics of Developmental and Neuropsychiatric Disorders, Ministry of Education, Shanghai Jiao Tong University, Shanghai, 200240 China; 2 Department of Joint Surgery, Shanghai East Hospital, School of Medicine, Tongji University, Shanghai, 200120 China; 3 Translational Medical Center for Stem Cell Therapy & Institute for Regenerative Medicine, Shanghai East Hospital, Tongji University School of Medicine, Shanghai, 200120 China

**Keywords:** MSC, Umbilical cord, scRNA-seq, Heterogeneity, Epithelia

## Abstract

Umbilical cord mesenchymal stem cells (UC-MSCs) have certain advantages over other MSCs and about 300 clinical trials have been registered using UC-MSCs to treat diseases such as osteoarthritis, autoimmune diseases, and degenerative disorders, yet, only limited success has been achieved. One reason is that in vitro expanded UC-MSCs show tremendous heterogeneity and their relationship to in vivo UC-MSCs remains unknown. Here, we investigated freshly isolated, uncultured UC-MSCs by single-cell RNA sequencing (scRNA-seq) and found two populations of UC-MSCs. Although UC-MSCs share many expressed genes and may have the same origin, they can be clearly separated based on differentially expressed genes including CD73 and other markers. Moreover, group 1 MSCs are enriched in expression of genes in immune response/regulatory activities, muscle cell proliferation and differentiation, stemness, and oxidative stress while group 2 MSCs are enriched with gene expression in extracellular matrix production, osteoblast and chondrocytes differentiation, and bone and cartilage growth. These findings suggest that UC-MSCs should be separated right after isolation and individually expanded in vitro to treat different diseases.

**Supplementary information:**

The online version contains supplementary material available at 10.1186/s13287-020-02055-1.

## Letter

Mesenchymal stem/stromal cells (MSCs) are a group of cells that can adhere to plastic surface and proliferate, express CD73, CD90, and CD105 but not CD34, CD45, CD11b, or HLA Class II, and can differentiate into osteoblast, chondrocyte, and adipocyte in vitro [1, 2]. They were first identified in the bone marrow and later detected in many tissues including adipose tissues, umbilical cord (Warton’s jelly) (UB-MSC), dermis, and placenta. Among MSCs of various sources, UC-MSCs have attracted much interest as these cells show low differentiation status, low immunogenicity, and easy to standardize [3]. Up to now, about 300 clinical trials have been registered using UC-MSCs to treat diseases such as osteoarthritis, autoimmune diseases, and degenerative disorders, yet, only limited success has been achieved [4]. MSCs may execute the therapeutic effects by immune suppression, differentiating into tissue cells, secretion of extracellular matrix, and providing pro-surviving signal molecules [1, 5]. However, the identity of UC-MSCs and their functions remain incompletely understood, thus hindering the clinical use of these cells.

**Fig. 1:**
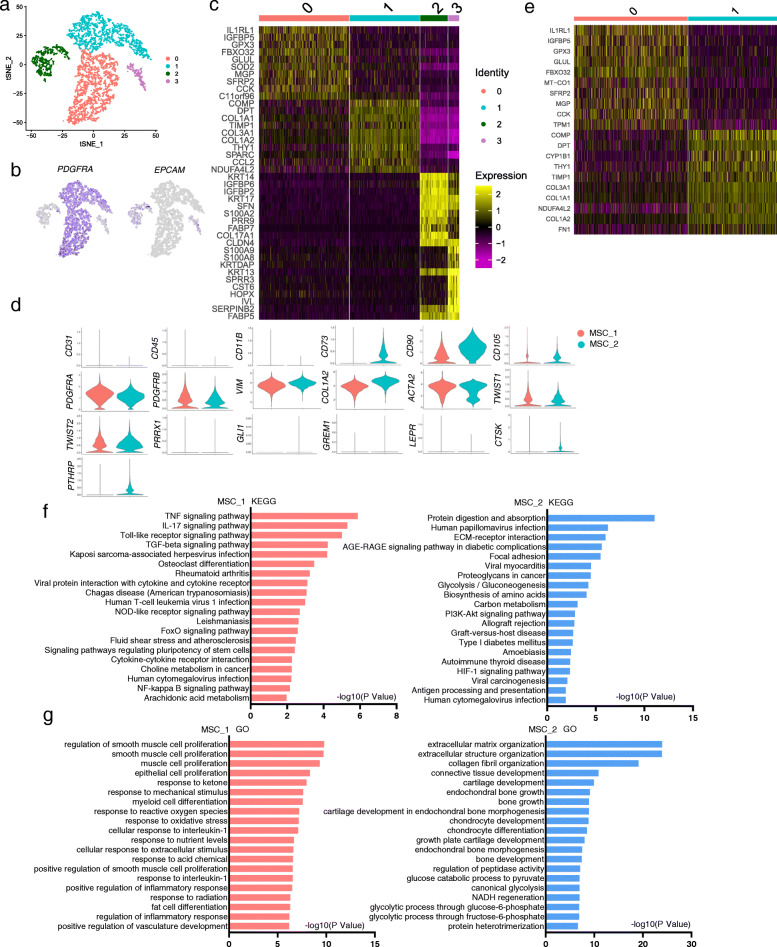
Clustering and characterization of uncultured umbilical cord cells. **a** t-NSE analysis of cells released from the Warton’s jelly of human umbilical cord. **b** Heatmap showing the top 10 differentially expressed signature genes in MSC and epithelial cell subgroups. **c** Expression of *PDGFRα* and *EPCAM* in MSC and epithelial cell subgroups. **d** Expression of selected key genes in the two UC-MSC subpopulations. **e** Heatmap showing the top 10 differentially expressed signature genes in the two MSC subpopulations. **f** KEGG analysis of the two UC-MSC subpopulations. **g** GO analysis of the two UC-MSC subpopulations

scRNA-seq has become a powerful tool to characterize tissue stem cells [6]. Previous studies have analyzed cultured UC-MSCs with scRNA-seq and found that UC-MSCs could be divided into 11 subgroups [7], which showed differences in expression of genes encoding extracellular matrix (ECM), protein process, and cell cycle-regulating proteins. In this study, we analyzed MSCs freshly isolated from Warton’s jelly of human umbilical cord and compared them to cultured UC-MSCs. We used a widely-used protocol to isolate human UC-MSCs (after removing blood vessels). We found that UC cells isolated with this protocol could adhere to plastic petri dishes and proliferate for at least 4 passages. Flow cytometry analysis showed that the cultured cells were negative for CD34, CD45, CD11b, and HLA-DR but positive for CD73, CD90, CD105, and CD44 (Supplementary Figure S1a). They also expressed very low levels of CD146 and CD200 (Supplementary Figure S1a). They could differentiate into osteoblasts, chondrocytes, or adipocytes in vitro (Supplementary Figure S1b). Thus, these UC-MSCs meet the criteria of MSCs. We then carried out scRNA-seq on freshly isolated UC-MSCs. A total of 5330 cells were sequenced at the depth of 3800 genes per cell (Fig. 1a). t-SNE analysis revealed two populations of epithelial cells and two populations of MSCs with the former expressing epithelial cell signature genes, e.g., *EPCAM*, *KRT13*, *KRT14*, and *KRT17*, and the latter expressing mesenchymal signature genes, e.g., *PDGFRA*, *COL1A1*, *COL1A2*, and *COL3A1* (Fig. 1b–d).

The two epithelial subpopulations have different features with one group expressing ECM genes and the other expressing development-related genes (Supplementary Figure S2A and B), suggesting that group 1 may represent epithelial progenitors while group 2 may represent the amniotic or cord-lining epithelia. Interestingly, both groups express CD29 and CD44, which are believed to be stem/progenitor cell markers, consistent with the primitive nature of cells of embryonic tissues. We then focused on the MSCs. The gene expression profiles of the two MSC subpopulations were similar (Fig. 1b and d), and both groups expressed *PDGFRA*, *VIM*, *COL1A2*, and *ACTA2* (Fig. 1d and supplementary Figure S3), suggesting that the two MSC groups might have the same origin. However, 176 genes were differentially expressed (Fig. 1e and supplementary Table S2), suggesting that they may have distinct functions.

Examination of cell surface marker gene expression revealed that neither group expressed *CD31*, *CD34*, *CD45*, or *CD11b* (Fig. 1d and supplementary Figure S4). They expressed low levels of *CD73*, *CD90*, and *CD105*, the common MSC markers, although *CD73* was mainly expressed in group 1 MSCs (Fig. 1d and supplementary Figure S4). Moreover, they expressed low levels of *CD200* but not *CD106* or *CD146* (Fig. 1d and supplementary Figure S4). Thus, the surface marker expression pattern of uncultured UC-MSCs is different from cultured UC-MSCs.

MSCs in the bone marrow are believed to be skeletal stem cells, which can be marked by PRRX1, TWIST2, LEPR, GREMLIN1, GLI1, PTHRP, and/or CTSK [8]. We analyzed the expression of these markers and found that both UC-MSC groups expressed *TWIST2* (and *TWIST1*) but not *PRRX1*, GLI1, GREMLIN 1, or LEPR, and a portion of group 2 cells expressed *PRRX1*, *PTHRP*, or *CTSK* (Fig. 1d and supplementary Figure S5a). In addition, there is evidence that MSCs are pericytes although later studies produced conflicting results [9, 10]. Our scRNA-seq data showed that UC-MSCs expressed some of the pericyte markers including *DESMIN*, *CD13*, and *CD248* but not *NG2*, *ANG1/2*, or *RGS5* (Supplementary Figure S5b), suggesting that UC-MSCs are not typical pericytes. The lack of pericytes can be explained by removal of the blood vessels and associated cells during MSC isolation.

KEGG pathway analysis revealed that group 1 UC-MSCs were enriched with TNFα, IL17, TLR, TGFβ, infection, NOD, NF-κB, and PGE pathways, many of which are immune-related (Fig. 1f and Supplementary Figure S6a-c). These pathways drive the expression of chemokines and immunomodulatory including PGE2, suggesting that group 1 MSCs may play a role in immune response and/or regulation. In addition, group 1 cells were enriched in the expression of genes in controlling pluripotency (Fig. 1f), whereas group 2 cells were enriched in the expression of genes in protein metabolism, extracellular matrix, and glucose and amino acid metabolism pathways (Fig. 1f). These results suggest that the two UC-MSC groups may have different functions.

Gene Ontology enrichment analysis revealed that group 1 MSCs expressed genes in biological activities including inflammation, muscle proliferation, cell differentiation, and oxidative stress response while group 2 cells expressed genes enriched in ECM synthesis, bone and cartilage growth, and glucose metabolism (Fig. 1g), confirming that the two UC-MSC subpopulations may have different functions.

This study shows that the standard UC-MSC isolation protocol also yields epithelial cells, which can be removed by FACS sorting based on their cell surface markers (CD24^+^CD44^+^PTHRP^+^) (Supplementary Figures S4-S5). More importantly, we find that UC-MSCs can be divided into two subpopulations based on differentially expressed genes especially cell surface markers such as CD73. Group 1 MSCs have features that are reminiscent of the therapeutic activities of MSCs including immunomodulation, pro-survival, and differentiation potentials, whereas group 2 MSCs have features suggesting that they are at a more differentiated state than group 1 MSCs and may be useful for repairing degenerated cartilage.

While this study identifies two subpopulations in freshly isolated UC-MSCs, a recent study shows that cultured human UC-MSCs can be divided into 11 groups. KEGG and GO analyses show that none of these 11 subpopulations expresses immunomodulatory genes [7]. These findings, taken together, suggest that freshly isolated UC-MSCs give rise to more subpopulations during in vitro expansion, and moreover, these cells may lose their original gene expression patterns and activities. It will be interesting to compare the therapeutic effects of the two uncultured UC-MSC subpopulations against cultured cells on osteoarthritis and autoimmune disorders. Moreover, right culture conditions are needed to maintain pluripotency and the major features of the UC-MSC subpopulations for future cell-based therapy.

## Supplementary Information

**Additional file 1.** Methods used in this study.

**Additional file 2: Supplementary Table S1.** List of the top 50 genes differentially expressed in the two epithelial subpopulations.

**Additional file 3: Supplementary Table S2.** List of the top 50 genes differentially expressed in the two MSC subpopulations.

**Additional file 4: Supplementary Figure S1.** Cultured human UC-MSCs show major MSC features. a. Flow cytometry analysis of cell surface markers on the cultured UC-MSCs. b. In vitro differentiation potentials of human UC-MSCs. Histochemical staining was performed to assess the differentiation into osteoblasts, chondrocytes, and adipocytes. Scale bar: 50 μm.

**Additional file 5: Supplementary Figure S2.** Characterization of uncultured umbilical epithelial cells. a. Heatmap showing differentially expressed signature genes in the two epithelial cell subpopulations. b. KEGG and GO analysis of the two epithelial cell subpopulations.

**Additional file 6: Supplementary Figure S3.** Expression of fibroblast/stromal marker genes in group 1 and 2 UC-MSCs.

**Additional file 7: Supplementary Figure S4.** Expression of cell surface marker genes in group 1 and 2 UC-MSCs.

**Additional file 8: Supplementary Figure S5.** Expression of skeletal stem cell (A) and pericyte (B) markers genes in the two UC-MSC subpopulations.

**Additional file 9: Supplementary Figure S6.** IL17, TGF, and TNF pathway were activated in group 1 UC-MSCs. Genes in red are up-regulated.

## Data Availability

The scRNA-seq data have been deposited into NCBI with a project number “PRJNA643879”.

## Abbreviations

MSC: Mesenchymal stem/stromal cells
UC: Umbilical cord
scRNA-seq: Single-cell RNA sequencing
KEGG: Kyoto Encyclopedia of Genes and Genomes
GO: Gene Ontology
