# Supplementary material for: Single-cell transcriptome analysis of uncultured human umbilical cord mesenchymal stem cells
Source: Stem Cell Res Ther. 2021 Jan 7;12:25. doi: 10.1186/s13287-020-02055-1 (PMC7791785; doi:10.1186/s13287-020-02055-1)
Supplement: Supplementary file 1 — Additional file 1. Methods used in this study. [file 13287_2020_2055_MOESM1_ESM.docx]

**Methods**

**Cell isolation and culture**

The umbilical cord (UC) was cut into 5-6-cm-long pieces that were rinsed with phosphate-buffered saline (PBS) to remove the blood. After the blood vessels were removed, the UC pieces were cut into smaller fragments (approximate 2 mm in diameter). The minced cord was digested with 1 mg/ml type II collagenase (Sigma) for 1.5 hrs and then with 0.125% Trypsin (Gibco) for 20 mins. The digested tissue was successively filtered through 70-μm strainers (BD Biosciences) and then centrifuged at 500g for 5 min at 4^o^C. The cell pellet was resuspended with PBS containing 1% bovine serum albumin (Sigma) to a concentration of 1 million/ml cells for single-cell sequencing. Some of the cells were cultured with DMEM/F12 complete medium containing 10% FBS, 100 mg/ml penicillin, and 100 mg/ml streptomycin for further studies.

**10X Illumina Single-Cell RNA Sequencing**

The cell suspension was loaded into Chromium microfluidic chips with 3’v3 chemistry and barcoded with a 10X Chromium Controller (10X Genomics). RNA from the barcoded cells was subsequently reverse-transcribed and sequencing libraries constructed with reagents from a Chromium Single Cell 3’ v3 reagent kit (10X Genomics) following the manufacturer’s instructions. Sequencing was performed with Illumina NovaSeq 6000 (Illumina).

**Single-cell RNA analysis**

Raw reads were demultiplexed and mapped to the human reference genome by Cell Ranger (10X Genomics) pipeline using default parameters. The generated gene-cell expression matrice was used for the subsequent analysis in R version 3.6.1 using Seurat

version 3.1.5 [[11](#_ENREF_1)]. “Cells” fit any of the following criteria were excluded: <1000 expressed genes, <2500 UMIs (unique molecular identifiers), >5 UMIs mapped to mitochondria. Filtered cells were used for downstream graph-based clustering and t-SNE visualization. Different expression genes (DEGs) in each cluster were identified with the function “FindAllMarkers” in Seurat. “DoHeatmap” function in Seurat was used to obtain heatmap figures. “FeaturePlot” and “VlnPlot” functions in Seurat were used for the visualization of Specific genes’ expression.

**GO or KEGG pathway enrichment analysis.**

DEGs were mapped to the GO database or KEGG database and the enrichment analysis of GO term or KEGG pathway was performed using the function Clusterprofiler in R package [[12](#_ENREF_2)].

***In vitro* differentiation of human UC-derived MSCs**

The protocols for MSC differentiation experiments were previously reported [[13](#_ENREF_3)].

For osteoblastic differentiation, UC-derived MSCs were seeded at 5 × 10^4^/well in 12-well plates. 24 hrs later, the cells were switched into an osteogenic medium of DMEM/F12 complete medium containing 10 mM β-glycerol phosphate and 50 μg/ml ascorbic acid for 7 days. The cells were then fixed in 4% paraformaldehyde and stained for ALP using an Alkaline Phosphatase Kit (Sigma-Aldrich).

For adipocyte differentiation, UC-derived MSCs were seeded at 1 × 10^5^/well in 12-well plates and cultured in DMEM/F12 complete medium containing 100 nM dexamethasone and 5 μM insulin for 2 weeks. The cells were then fixed and stained with Oil red O solution.

For chondrogenesis assays, UC-derived MSCs were suspended at a concentration of 1.6 × 10^7^ cells/ml. We generated micro-mass cultures by seeding 10 µl droplets of cell suspension at the center of 12-well plates. Cells were allowed to attach for 2 hr before adding DMEM/F12 complete medium. 24 hrs later, the medium was replaced with a chondrogenic medium of DMEM/F12 complete medium containing 100 nM dexamethasone, 10 ng/ml TGFβ1, and 1 μM ascorbate-2-phosphate. Cultures were maintained for 21 days and then stained with Alcian Blue.

**Flow-Cytometric analysis**

All the FACS antibodies were purchased from Biolegend. Cultured UC-derived MSCs were collected and stained with antibodies against CD45-APC, CD11b-FITC, HLA-DR-FITC, CD34-FITC, CD105-FITC, CD44-FITC, CD73-FITC, CD90-FITC, CD146-FITC, and CD200-APC respectively for 30 min at room temperature. Then, FACS analysis was performed on CytoFlex S (Beckman). The FACS data were analyzed by Flowjo 10.5.3.

**References**

11. Butler A, Hoffman P, Smibert P, Papalexi E, Satija R: **Integrating single-cell transcriptomic data across different conditions, technologies, and species**. *Nat Biotechnol* 2018, **36**(5):411-420.

12. Yu GC, Wang LG, Han YY, He QY: **clusterProfiler: an R Package for Comparing Biological Themes Among Gene Clusters**. *Omics* 2012, **16**(5):284-287.

13. Deng Q, Li P, Che M, Liu J, Biswas S, Ma G, He L, Wei Z, Zhang Z, Yang Y *et al*: **Activation of hedgehog signaling in mesenchymal stem cells induces cartilage and bone tumor formation via Wnt/beta-Catenin**. *Elife* 2019, **8**.
