# Supplementary material for: Single-cell transcriptome analysis of uncultured human umbilical cord mesenchymal stem cells
Source: Stem Cell Res Ther. 2021 Jan 7;12:25. doi: 10.1186/s13287-020-02055-1 (PMC7791785; doi:10.1186/s13287-020-02055-1)
Supplement: Supplementary file 3 — Additional file 3: Supplementary Table S2. List of the top 50 genes differentially expressed in the two MSC subpopulations. [file 13287_2020_2055_MOESM3_ESM.docx]

**Supplementary Table S2. List of the top 50 genes differentially expressed in the two MSC subpopulations.**

**
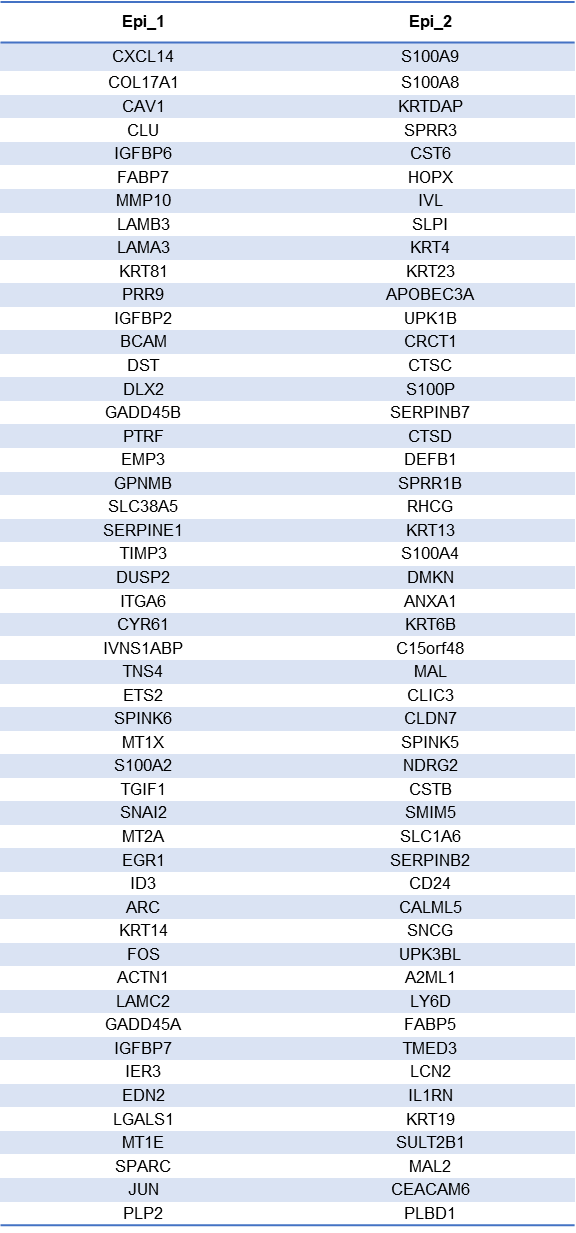
**
