# Supplementary material for: Single-cell transcriptome analysis of uncultured human umbilical cord mesenchymal stem cells
Source: Stem Cell Res Ther. 2021 Jan 7;12:25. doi: 10.1186/s13287-020-02055-1 (PMC7791785; doi:10.1186/s13287-020-02055-1)
Supplement: Supplementary file 4 — Additional file 4: Supplementary Figure S1. Cultured human UC-MSCs show major MSC features. a. Flow cytometry analysis of cell surface markers on the cultured UC-MSCs. b. In vitro differentiation potentials of human UC-MSCs. Histochemical staining was performed to assess the differentiation into osteoblasts, chondrocytes, and adipocytes. Scale bar: 50 μm. [file 13287_2020_2055_MOESM4_ESM.docx]

ALP (osteoblast)

Alcian blue (chondrocyte )

Oil red (adipocyte )


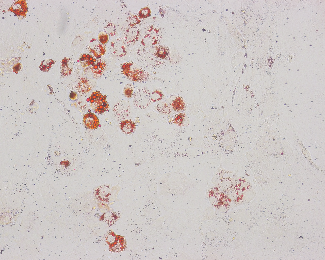

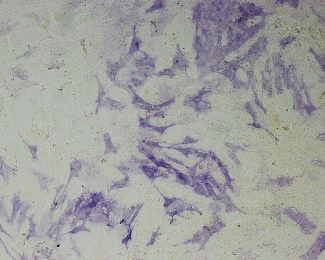

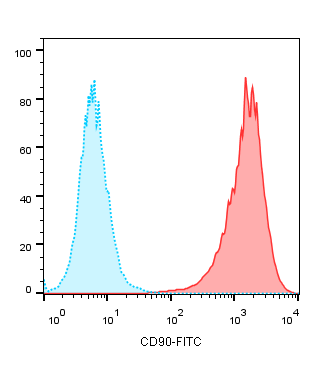


CD90


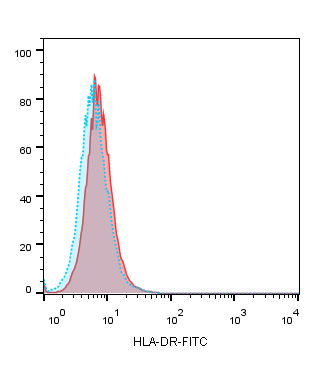


HLA-DR


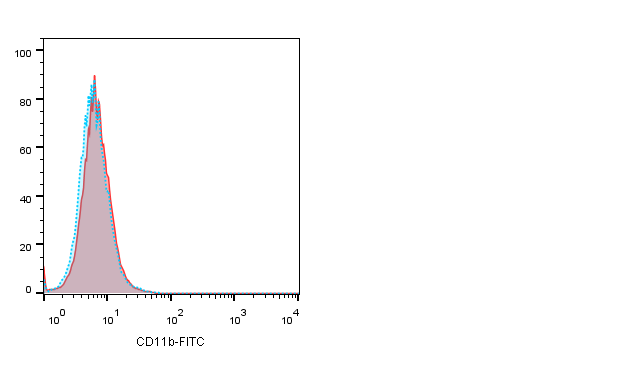


CD11b


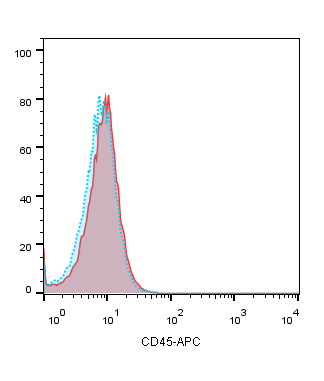


CD45


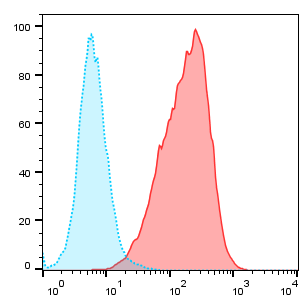


CD105


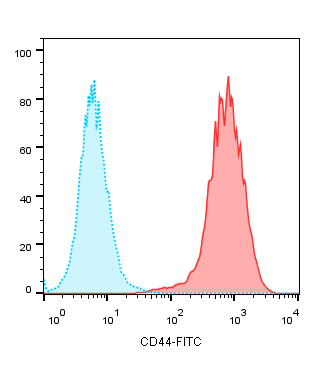


CD44


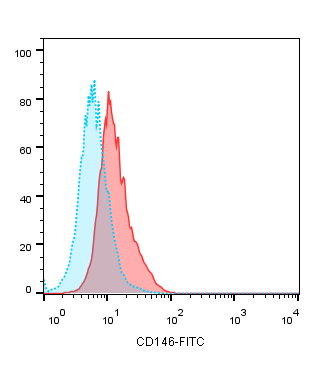


CD146


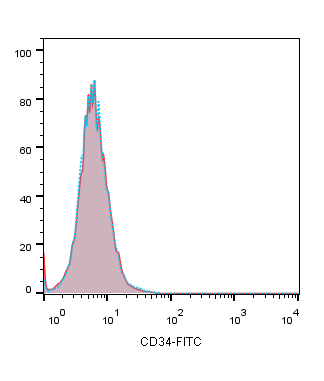


CD34


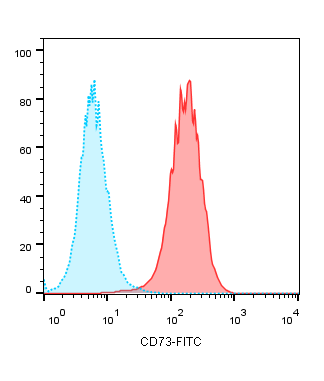


CD73.


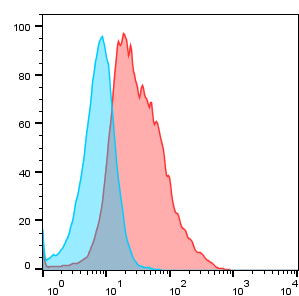


CD200

a

b


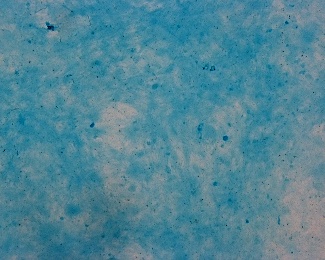


Supplementary Fig. S1. Cultured human UC-MSCs show major MSC features.

a. Flow cytometry analysis of cell surface markers on the cultured UC-MSCs.

b. In vitro differentiation potentials of human UC-MSCs. Histochemical staining was performed to assess the differentiation into osteoblasts, chondrocytes, and adipocytes. Scale bar: 50 μm.
