# Supplementary material for: Single-cell transcriptome analysis of uncultured human umbilical cord mesenchymal stem cells
Source: Stem Cell Res Ther. 2021 Jan 7;12:25. doi: 10.1186/s13287-020-02055-1 (PMC7791785; doi:10.1186/s13287-020-02055-1)
Supplement: Supplementary file 5 — Additional file 5: Supplementary Figure S2. Characterization of uncultured umbilical epithelial cells. a. Heatmap showing differentially expressed signature genes in the two epithelial cell subpopulations. b. KEGG and GO analysis of the two epithelial cell subpopulations. [file 13287_2020_2055_MOESM5_ESM.docx]

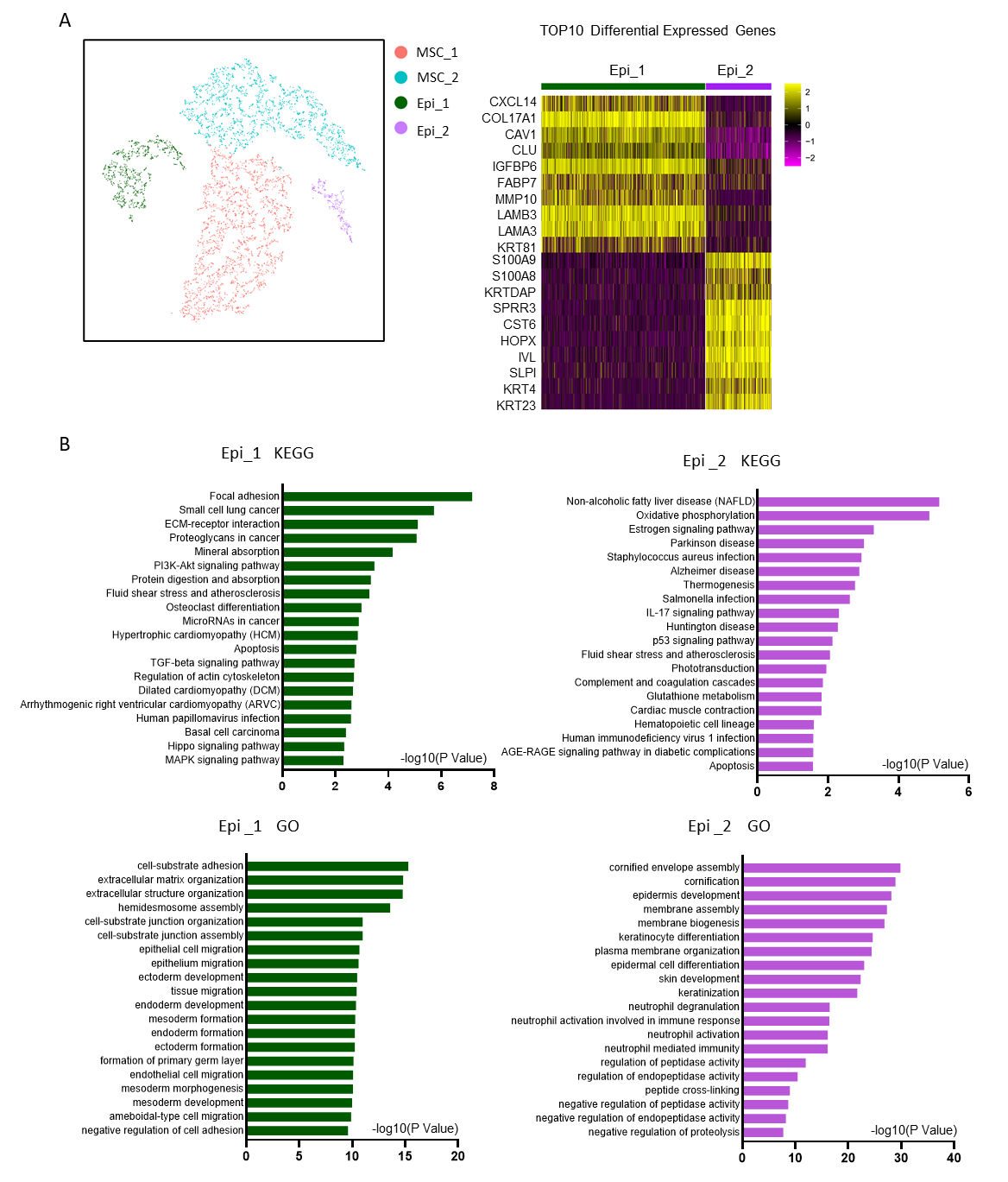


Supplementary Fig. S2. Characterization of uncultured umbilical epithelial cells.

a. Heatmap showing the differentially expressed signature genes in the two epithelial cell subpopulations.

b. KEGG and GO analysis of the two epithelial cell subpopulations.
