# Supplementary figures and images for: Single-cell transcriptome analysis of uncultured human umbilical cord mesenchymal stem cells
Source: Stem Cell Res Ther. 2021 Jan 7;12:25. doi: 10.1186/s13287-020-02055-1 (PMC7791785; doi:10.1186/s13287-020-02055-1)

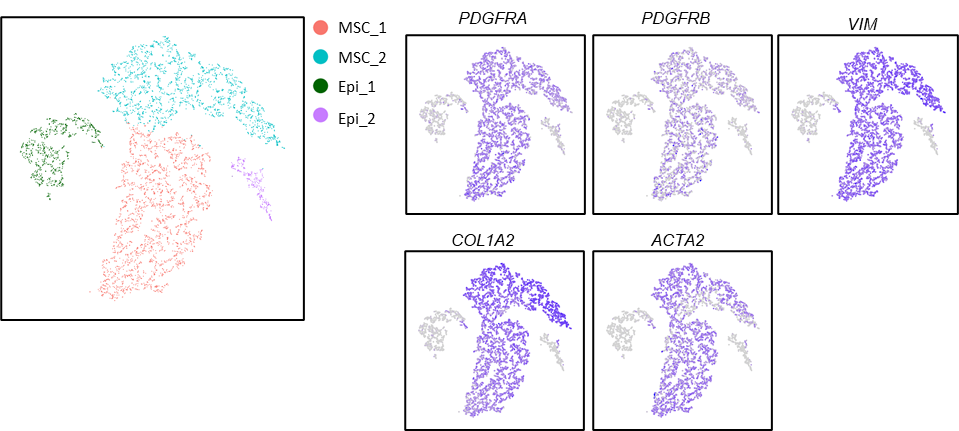


Supplementary Fig. S3. Expression of fibroblast/stromal marker genes in group 1 and 2 UC-MSCs.

Supplement: Supplementary file 6 — Additional file 6: Supplementary Figure S3. Expression of fibroblast/stromal marker genes in group 1 and 2 UC-MSCs. [file 13287_2020_2055_MOESM6_ESM.docx]
