# Supplementary material for: Single-cell transcriptome analysis of uncultured human umbilical cord mesenchymal stem cells
Source: Stem Cell Res Ther. 2021 Jan 7;12:25. doi: 10.1186/s13287-020-02055-1 (PMC7791785; doi:10.1186/s13287-020-02055-1)
Supplement: Supplementary file 7 — Additional file 7: Supplementary Figure S4. Expression of cell surface marker genes in group 1 and 2 UC-MSCs. [file 13287_2020_2055_MOESM7_ESM.docx]

*CD29*

*CD31*

*CD45*

*CD11B*

*CD105*

*CD106*

*CD200*

*CD24*

*CD44*

*CD73*

*CD90*

MSC_1

Epi_1

Epi_2

MSC_2


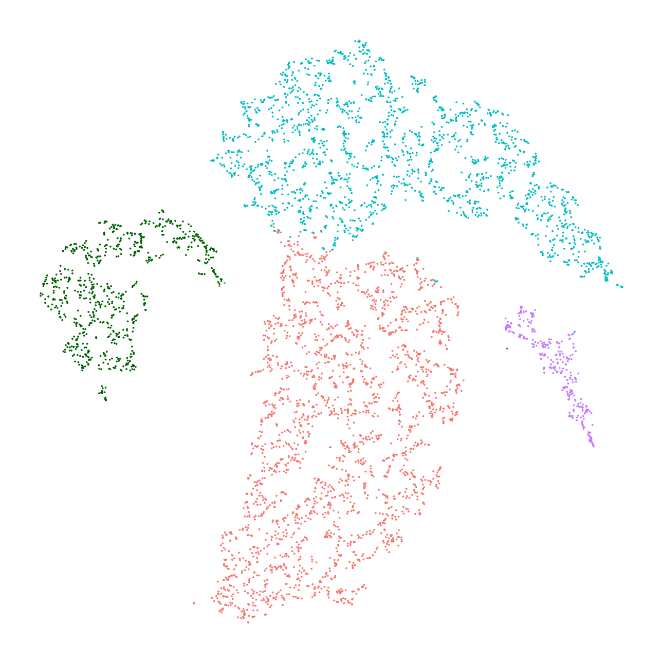

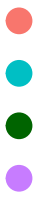

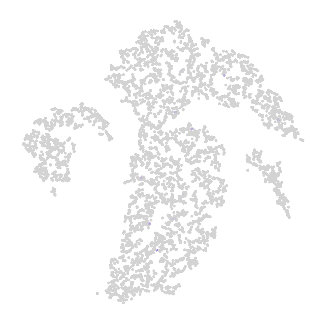

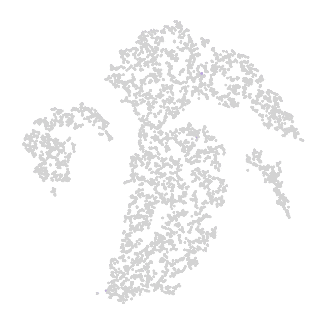

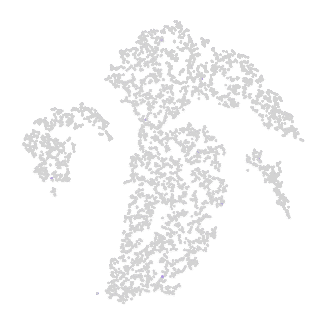

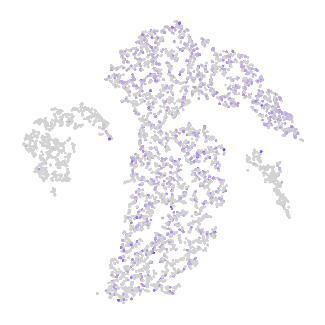

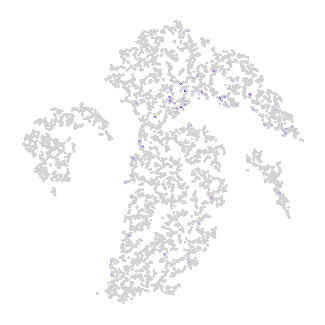

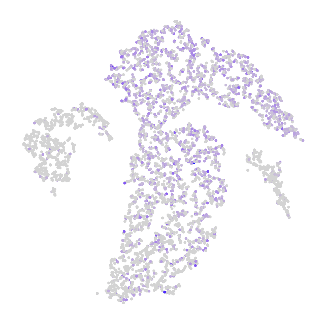

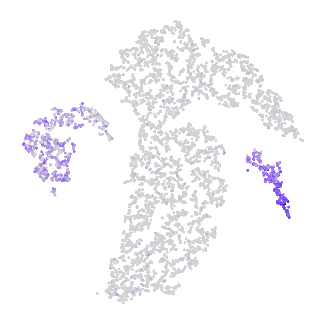

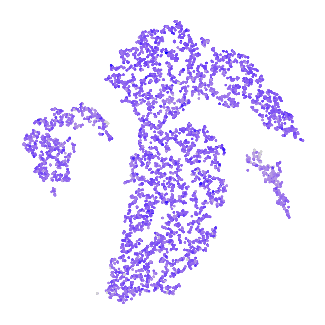

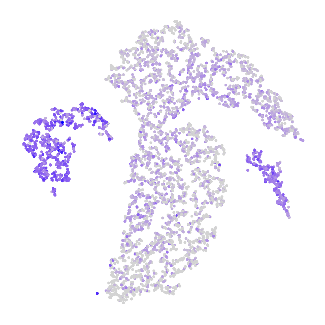

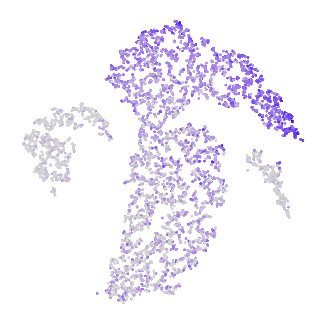

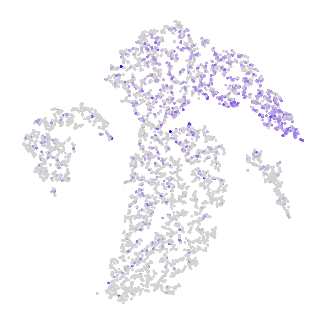


*CD146*


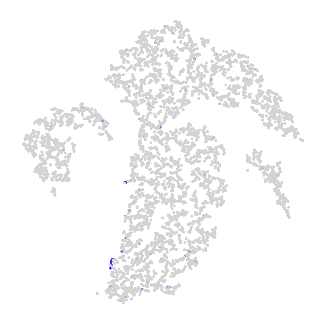


Supplementary Fig. S4. Expression of cell surface marker genes in group 1 and 2 UC-MSCs.
