# Supplementary material for: Single-cell transcriptome analysis of uncultured human umbilical cord mesenchymal stem cells
Source: Stem Cell Res Ther. 2021 Jan 7;12:25. doi: 10.1186/s13287-020-02055-1 (PMC7791785; doi:10.1186/s13287-020-02055-1)
Supplement: Supplementary file 8 — Additional file 8: Supplementary Figure S5. Expression of skeletal stem cell (A) and pericyte (B) markers genes in the two UC-MSC subpopulations. [file 13287_2020_2055_MOESM8_ESM.docx]

a

*TWIST1*

*TWIST2*

*PRRX1*

*GLI1*

*GREM1*

*LEPR*

*CTSK*

*PTHRP*

*NG2*

*RGS5*

*DES*

*CD13*

*ANG1*

*ANG2*

*CD248*

MSC_1

Epi_1

Epi_2

MSC_2


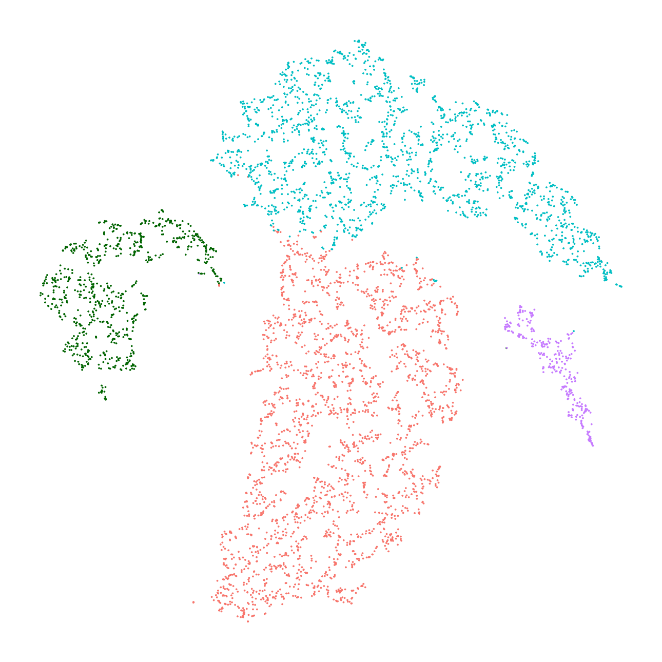

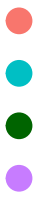

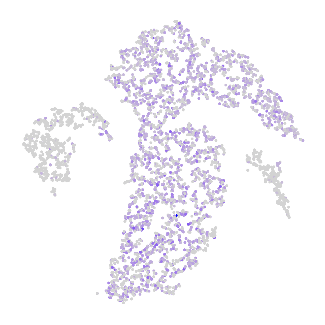

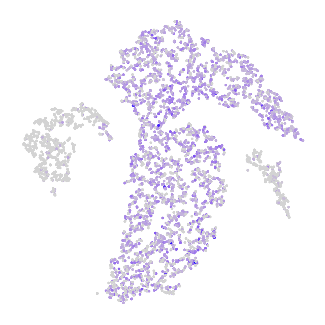

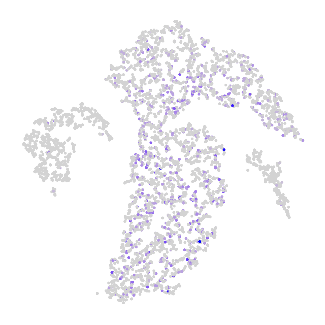

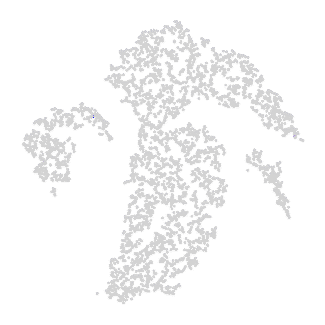

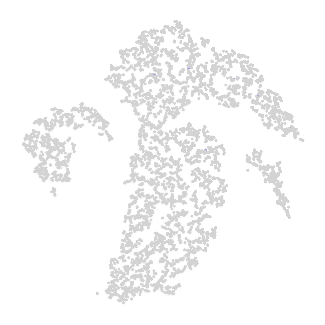

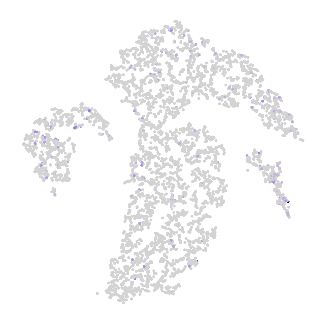

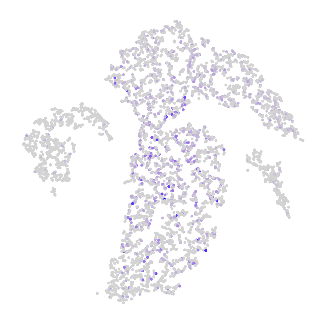

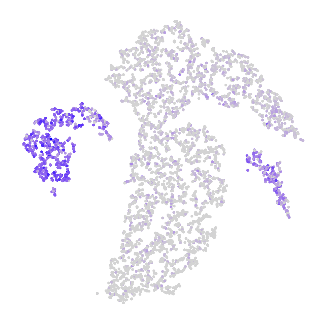

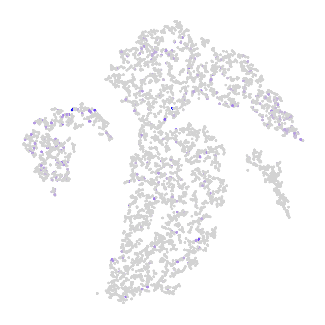

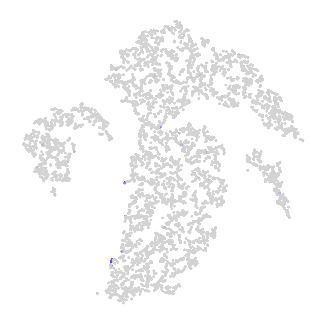

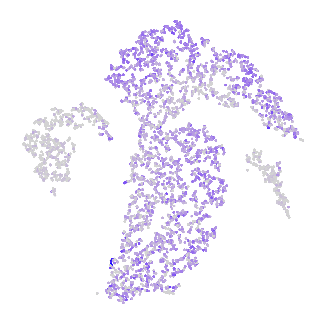

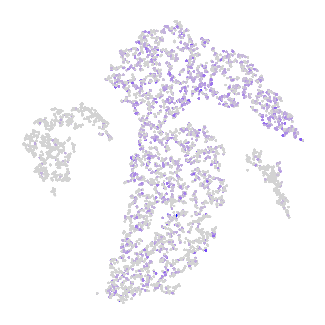

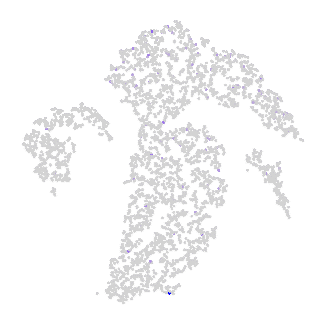

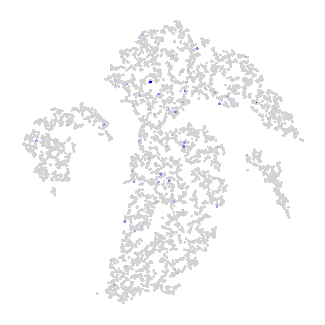

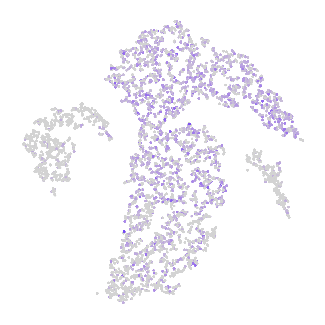


b

Supplementary Fig. S5. Expression of skeletal stem cell (A) and pericyte (B) markers genes in the two UC-MSC subpopulations.
