# Supplementary material for: Single-cell transcriptome analysis of uncultured human umbilical cord mesenchymal stem cells
Source: Stem Cell Res Ther. 2021 Jan 7;12:25. doi: 10.1186/s13287-020-02055-1 (PMC7791785; doi:10.1186/s13287-020-02055-1)
Supplement: Supplementary file 9 — Additional file 9: Supplementary Figure S6. IL17, TGF, and TNF pathway were activated in group 1 UC-MSCs. Genes in red are up-regulated. [file 13287_2020_2055_MOESM9_ESM.docx]

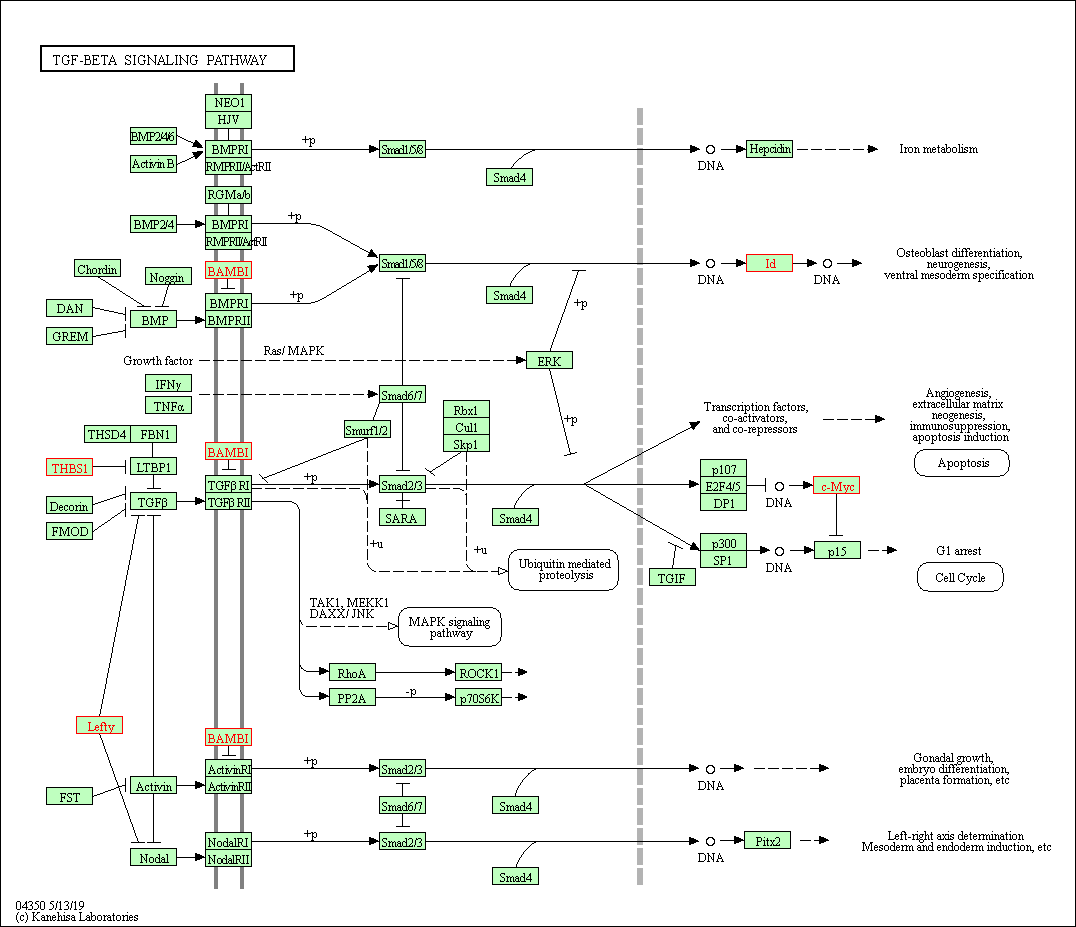

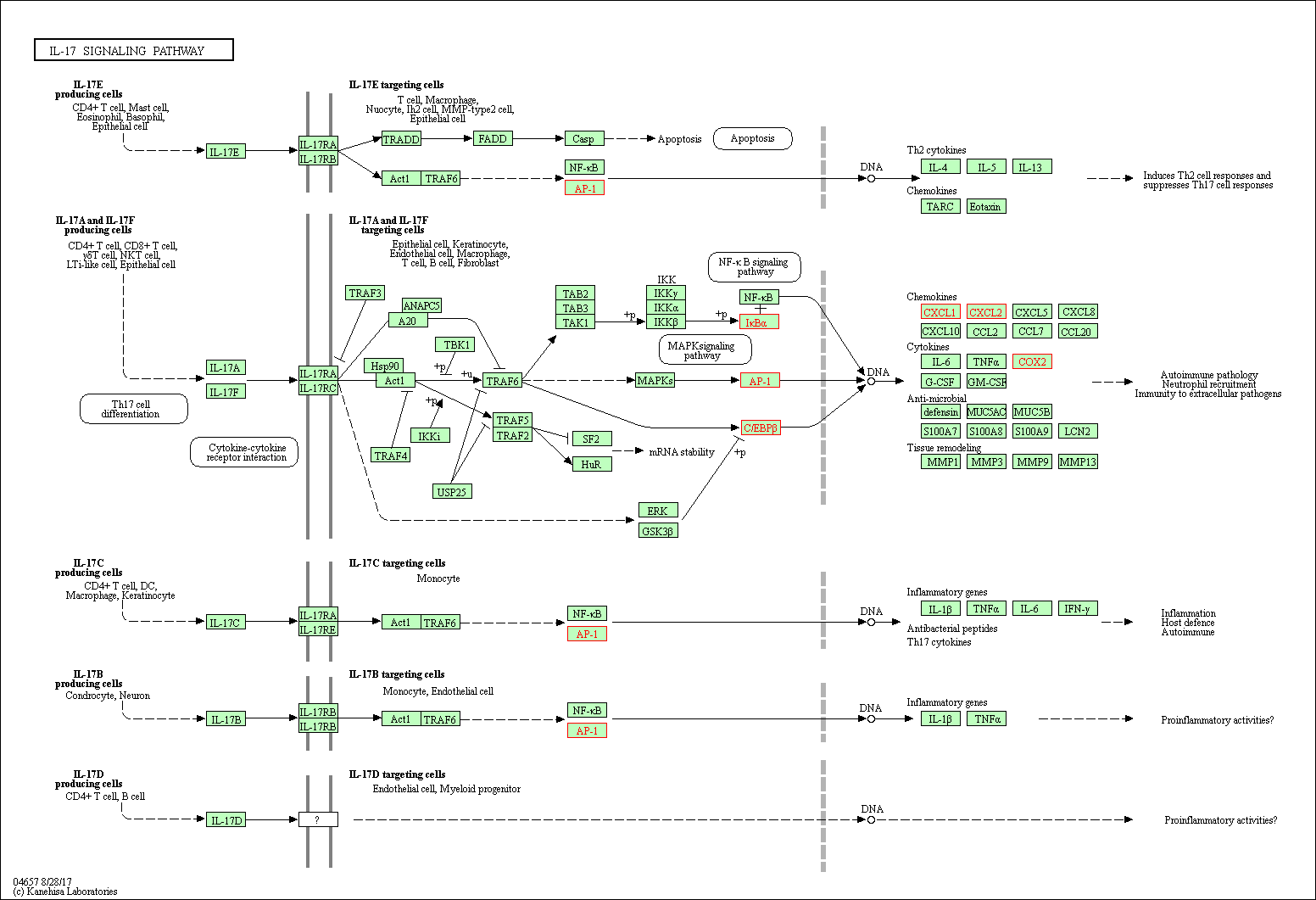

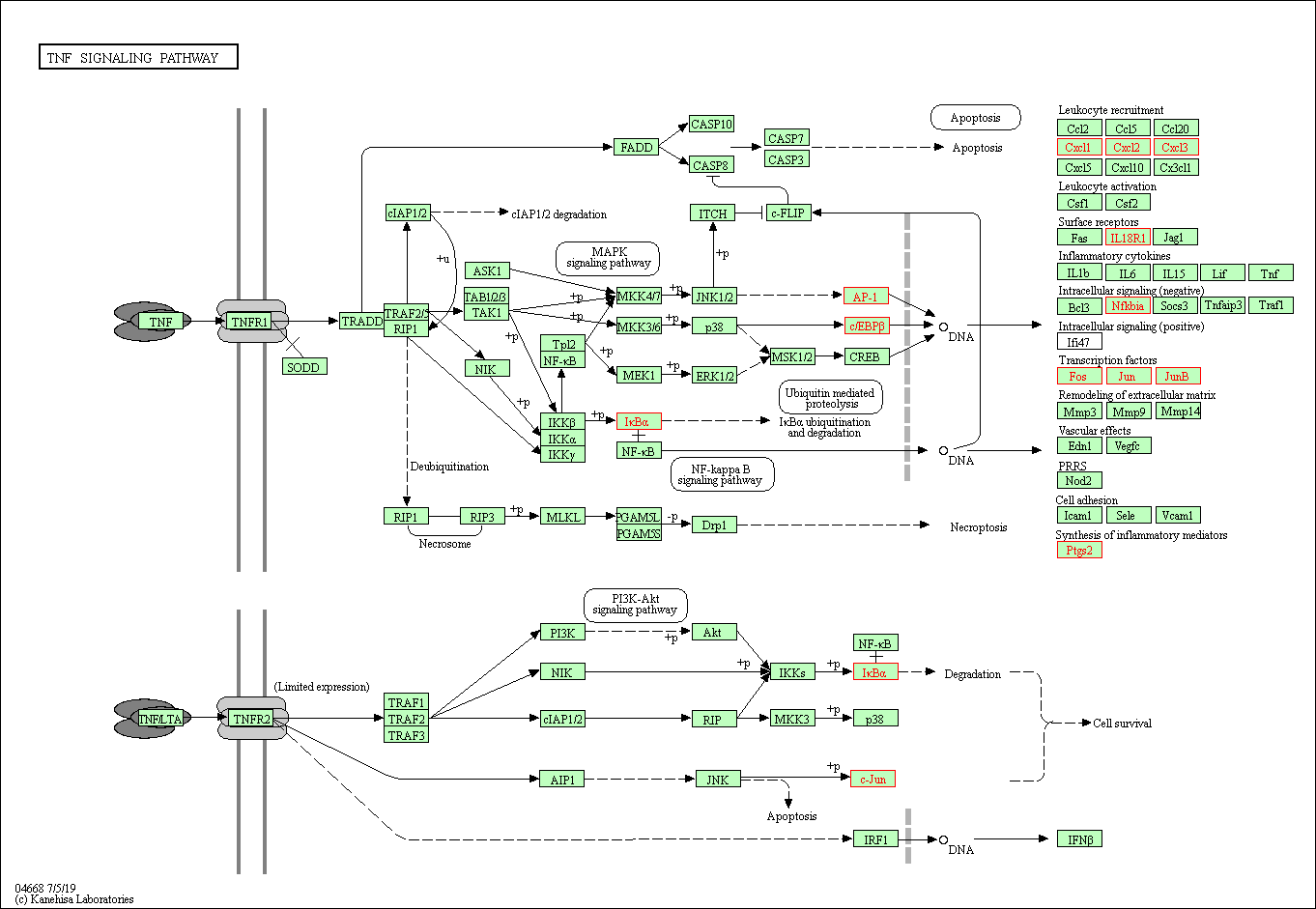


a

b

c

Supplementary Fig.6. IL17, TGFβ, and TNFα pathway were activated in group 1 UC-MSCs. Genes in red are up-regulated.
